# Effect of Age, Gender, Food Intake, Obesity, and Smoking on Serum Levels of Prolactin in Healthy Adults

**DOI:** 10.3390/jpm14090905

**Published:** 2024-08-27

**Authors:** Munther S. Momani, Ahmad Al Tarawni, Yazan M. Momani, Sara Rahhal, Ibrahim Elhaj, Dania Al-Halhouli, Hussam Alhawari

**Affiliations:** 1 Internal Medicine Department, School of Medicine, University of Jordan, Queen Rania Street, Amman 11942, Jordan; 2 School of Medicine, University of Jordan, Queen Rania Street, Amman 11942, Jordansar0180690@ju.edu.jo (S.R.); abr0182201@ju.edu.jo (I.E.);

**Keywords:** age, gender, body weight, smoking, feeding, prolactin hormone

## Abstract

Objective: The objective of this study was to investigate the effect of age, gender, body mass index (BMI), food intake, and smoking on serum prolactin (PRL) levels among healthy adults. Methods: In total, 100 healthy adults (46 males and 54 females) aged ≥18 years were recruited. Serum PRL levels were measured after a 9 h overnight fast, 1 h postprandially (early), and 3 h postprandially (late). Results: Mean PRL levels were significantly higher in females than males in the fasting, early, and late postprandial states. PRL levels were suppressed significantly after food intake in both genders. Mean PRL levels were significantly higher in younger individuals (<30 years) in the fasting and the early postprandial states compared to older individuals (≥30 years) and suppressed significantly after food intake in younger individuals only. Mean PRL levels were not significantly different between individuals with a normal BMI (<25 kg/m^2^) compared to those overweight and with obesity (BMI ≥ 25 kg/m^2^); however, PRL levels were significantly suppressed after food intake only in subjects with a normal BMI. Mean PRL levels did not differ significantly according to smoking status. PRL levels were suppressed significantly after food intake in nonsmokers only. Conclusion: Our data suggest that age, gender, and food intake should be considered when the serum PRL level is measured in clinical practice.

## 1. Introduction

Prolactin (PRL) is a pleiotropic hormone synthesized and secreted by lactotroph cells in the anterior pituitary gland. The main function of PRL is milk production in mammals. The PRL receptor is expressed in various tissues and cells such as the endometrium, prostate, pancreatic islets, and adipocytes. Prolactin has been suggested to play a role in metabolism, energy homeostasis, immune modulation, and regulation of hair, skin, and bone [1,2,3,4,5,6]. Prolactin secretion can be initiated by multiple hormones including estrogens and thyrotropin-releasing hormone [7,8]. Drug-naïve patients and pregnant or lactating women display higher basal PRL levels compared to males and non-pregnant females. A pituitary adenoma or drug side effect, among other etiologies, can cause an abnormal PRL increase in males [9]. Serum PRL levels are used clinically to evaluate disorders involving the pituitary gland and occasionally to detect mass lesions adjacent to and compressing the gland and/or its stalk.

In standard clinical practice, no strict prerequisite conditions are implemented for a routine serum PRL measurement in most clinical settings. The Pituitary Society Guidelines issued in 2023 are lenient and do not give specific recommendations for the timing of prolactin measurement and its relation to food intake [10].

It has been suggested that variables such as age, sex, body weight, and feeding status affect the serum PRL level. However, studies in this regard remain limited, contain small sample sizes, and the results are conflicting [8,11,12,13,14,15]. The main aim of this study was to determine the influence of age, gender, BMI, food intake, and smoking on the levels of serum PRL among a larger population of healthy subjects.

## 2. Methods

### 2.1. Recruitment of Subjects

This cross-sectional study included one hundred healthy volunteers aged 18 years or older. The sample size was selected based on the results of a review of the relevant literature, as most similar studies included much smaller sample sizes. Subjects were selected from healthy consenting hospital employees and medical students. Subjects with known chronic medical illnesses, including those with PCOS, thyroid or pituitary disorders, and those taking any regular medications were excluded. In addition, subjects with baseline serum PRL, thyroid-stimulating hormone (TSH), or free thyroxine levels falling outside the normal reference range of the assay used in the study were also excluded. None of our subjects consumed alcohol. Signed informed consent was obtained from all participants after an explanation of the study. Participants were not coerced in any way to enroll, nor were they under any pressure or obligation to volunteer or complete the study. Approval for the study was obtained from the institutional ethics committee at our university hospital.

### 2.2. Laboratory Measurements and Measurement Conditions

Blood samples were drawn at 08:00 h after a 9 h overnight fast. Participants were then asked to immediately have a breakfast of their choice. They were offered individualized breakfast choices including cereals, bread, cake, cheese, turkey breast, chicken, beef, and yogurt, etc. The participants were allowed to eat their usual foods without special restrictions to mimic real-life conditions. All subjects were observed to have a mixed meal of their choice. Beverages like coffee, tea, or juice were not accepted alone as breakfast. The participants took 10 to 15 min to finish their breakfast. A second blood sample was drawn at 09:00 h. The aim of sampling at 09:00 was to compare the fasting and early postprandial serum PRL levels. A third sample was taken at 11:00 (3 h postprandially) to determine the delayed effect of food intake on the measured hormone levels.

Participants were allowed to resume daily activities freely throughout the study period. They were allowed to undertake mild to moderate physical activity, such as walking, sitting, and standing, but vigorous exercise was not permitted. No previous preparation or dietary modification was required prior to the overnight fast. All blood samples were drawn from the antecubital vein while the subject was sitting. All blood samples were analyzed in one reference laboratory.

Serum TSH and free thyroxine levels were measured to exclude any effect of thyroid dysfunction on the serum PRL levels.

### 2.3. Assay Specifications

Serum PRL, TSH, and free thyroxine levels were measured using the fully automated MAGLUMI 2000 Plus chemiluminescence immunoassay (CLIA) analyzer (Snibe Co., Ltd., Shenzhen, China). Normal values for PRL according to this assay are 2.5–15.9 ng/mL for males and 3.1–23 ng/mL for females. The intra-assay coefficients of variation are 9.06% at 16.24 ng/mL and 5.13% at 49.52 ng/mL, and the standard deviation (SD) values are 1.47 and 2.54 ng/mL at 16.24 and 49.52 ng/mL, respectively. The inter-assay coefficient of variation is 8.76% at 16.52 ng/mL and the standard deviation (SD) is 1.46 ng/mL.

Normal values for TSH according to this assay are 0.4–4.5 mIU/mL. The intra-assay coefficient of variation is 3.26% with a standard deviation of 0.04 mIU/mL. The inter-assay coefficient of variation is 7.03% at a mean of 0.52 mIU/mL with a standard deviation (SD) of 0.04 mIU/mL. Normal values for free thyroxine according to this assay are 0.89–1.72 ng/dL. The intra-assay coefficient of variation is 7.24% with a standard deviation of 0.011 ng/dL. The inter-assay coefficient of variation is 8.85% at a mean of 1.40 ng/dL with a standard deviation (SD) of 0.12 pg/mL.

### 2.4. Other Definitions

Body mass index (BMI) was defined as weight measured in kilograms divided by squared height in meters. Smokers were defined as those who were smoking cigarette(s), pipe(s), cigar(s), or hookah(s) at the time of this study. Former smokers were considered as non-smokers.

### 2.5. Statistical Analysis

Data were analyzed using IBM SPSS Statistics for Windows, version 26.0 (IBM Corp., Armonk, NY, USA). We used the mean (±standard deviation) to describe continuous variables and percentages to describe categorical variables. The change in prolactin levels was analyzed using repeated measures analysis. The time * variable interaction terms were tested in the model. A *p*-value of less than 0.05 was considered statistically significant.

## 3. Results

A total of 100 healthy adult volunteers (46 men and 54 women) with a mean age of 29.2 (±9.7) years were recruited. There were 46 (46%) men, with a mean age of 28.5 (±9.0) years, and 54 (54%) women, with a mean age of 29.8 (±10.2) years. In all, 63 (63%) subjects were younger than 30 years, and 37 (37%) were 30 years or older. BMI was normal in 51 (51%) subjects and high in 49 (49%) subjects (Table 1).

The mean PRL level in the fasting state was 12.52 (7.79) ng/mL, while it decreased to 10.54 (4.95) ng/mL 1 h after food intake and dropped further 3 h after food intake to 9.54 (4.63) ng/mL. Changes after food intake were both significant compared to fasting, with *p* values of <0.001 and 0.02, respectively. Table 2 illustrates the mean serum PRL levels in the fasting and postprandial states in the gender, age, BMI, and smoking groups.

Mean PRL levels were significantly higher in females in the fasting, early, and late postprandial states compared to males. Mean PRL levels were significantly higher in younger individuals (<30 years) in the fasting and the early postprandial states compared to older individuals (≥30 years), but not in the late postprandial state. Mean PRL levels were not significantly different between individuals with a normal BMI (<25 kg/m^2^) in the fasting, early, and late postprandial states compared to those overweight and with obesity (BMI ≥ 25 kg/m^2^). The mean fasting, early, and late postprandial PRL levels did not differ significantly according to smoking status.

**Figure 1:**
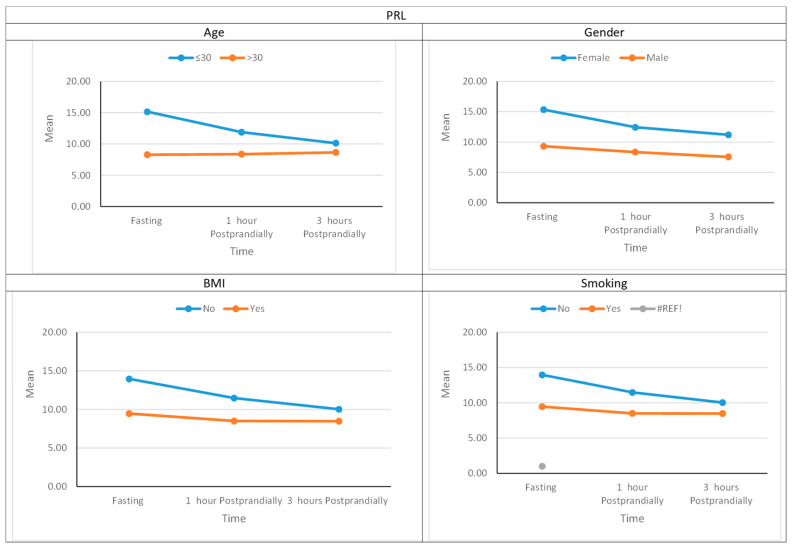
Prolactin levels over time in age, gender, BMI, and smoking subgroups.

The decrease in prolactin levels after food intake was significant in females in the early and late postprandial states compared to fasting. In males, the decrease in prolactin levels was significant only in the late postprandial state compared to fasting. Prolactin levels decreased significantly in both early and late postprandial states in the younger group (<30 years) and did not change significantly in the older group (≥30 years). Prolactin levels decreased significantly after food intake in both the early and late postprandial states in the group with normal BMI values, while the changes observed in prolactin levels in the overweight and obese group were not statistically significant. Prolactin levels were not statistically different between smokers and nonsmokers in the fasting and postprandial states; however, the nonsmokers’ group showed a significant decrease in prolactin levels after food intake in both the early and late postprandial states. The smokers’ group showed no significant change in prolactin levels after food intake. Figure 1 shows the mean prolactin levels over time in different subject subgroups.

## 4. Discussion

Serum PRL levels can influence the diagnosis of several pituitary gland disorders; thus, it is important for guidelines to provide clear recommendations on the ideal conditions for prolactin measurement and whether factors such as relation to the time of the day, nutritional status, gender, age, BMI, and smoking must be considered. Previous studies explored, among other factors, the effect of gender, age, obesity, and smoking on PRL levels. The results were inconclusive and, at times, conflicting.

We aimed in our current study to better clarify the effects of age, gender, BMI, food intake, and smoking on serum PRL levels. We believe that our study will clarify the inconsistent results reported in the literature and expand the current knowledge, as we present data from a larger sample size of healthy volunteers in conditions mimicking real-life eating experiences.

### 4.1. Gender and Serum PRL Level

The association between gender and PRL levels has been evaluated in many studies, with the vast majority of studies finding that PRL levels in females are higher at baseline than in males. This observation is in line with the use of sex-specific normal ranges for serum prolactin by prolactin assays.

A cross-sectional case–control study carried out by Del Cacho et al. [15] evaluated the differences in baseline PRL levels between first episode drug-naïve psychotic patients and healthy controls. It aimed to study whether there was a relationship between perceived stress levels and PRL levels, as well as to assess whether there were gender differences in the elevation of PRL. The study showed no significant differences in PRL levels between males and females in drug-naïve psychotic patients; this can be explained by the use of normalized prolactin levels (adjusted for age and sex) according to the authors [15]. Other studies, including the meta-analysis of González-Blanco et al., demonstrated that male patients had lower levels of PRL than female patients [16]. The same gender difference was seen in a prospective cross-sectional study conducted by Ernst et al. [17] in which women displayed significantly higher basal PRL levels than men (9.0 ± 4.8 vs. 7.9 ± 3.6 μg/L, *p* = 0.03) before as well as after the bariatric operation. The same trend was shown by Wagner et al. [18]. Our results showed a significantly higher baseline PRL level in women compared to men, which is consistent with what was reported by Roelfsema et al., Gonzalez-Blanco et al., Ernst et al., and Wagner et al. [7,16,17,18].

### 4.2. Age and Serum PRL Level

Few studies have reported on the association between age and PRL levels. Roelfsema et al. [7] reported on the effects of age, gender, and BMI on PRL levels. They reported that peak PRL levels decrease with advancing age. Another retrospective study by Wagner et al. concluded that PRL levels decline with age [18]. Iranmaesh et al. [6] also reported on declining PRL levels with aging in men and women, likely due to declining estrogen levels. This is consistent with our finding of lower baseline fasting PRL levels in individuals 30 years or older compared to those younger than 30 years. Since our subjects included very few postmenopausal women, we could not draw any conclusion regarding the effect of the menopause.

### 4.3. Food Intake and Serum PRL Level

Studies that evaluated the relationship of PRL levels to food intake are mostly limited in sample size and the results are conflicting. Some studies showed no change in prolactin levels in response to food intake. Plumelle et al. found no significant effect of food intake on prolactin levels; rather, the level was influenced by the timing of sampling as it decreased during the day [19]. Mei et al. also found no difference in prolactin levels in response to a Mediterranean diet combined with a low-carbohydrate diet vs. a low-fat diet [20]. Other studies also reported no change in PRL levels in response to food intake, including a study by Goettler et al., who reported unchanging PRL levels postprandially in a group of women with stage 1 breast cancer and the controls, although a significant rise in postprandial serum PRL levels was seen in stage II breast cancer patients, possibly due to increased levels of stress [21]. In a study of 393 premenopausal Japanese women, Tsuji et al. reported that there were no significant associations between the intake of unsaturated fat, total fat, dietary fiber, and PRL levels [22].

Other studies found a decrease in prolactin levels after food intake. Angelis et al., for example, reported a decrease in prolactin levels following the Mediterranean diet [23]. Hill et al. also reported that a vegetarian diet decreased prolactin levels [24].

Some studies reported increased PRL levels after food intake. Ishizuka et al. found increasing PRL levels after food, but their results were obtained from a sample of seven men only [25]. Carlson et al. also reported that protein significantly stimulated PRL secretion in both men and women [26]. In addition, Shultz et al. reported that PRL levels were positively correlated with dietary energy, protein, total and saturated fatty acids, and oleic acid [27]. Velázquez-Villegas et al. also reported increased PRL levels following a high-protein/low-carbohydrate diet in rats [28]. Table 3 summarizes the effect of food intake on serum PRL levels from the previously published literature.

Our study showed a decrease in serum PRL levels in the early and late postprandial states in the whole sample. The subjects in our study were healthy men and women who were allowed to choose from a variety of foods; thus, it was a “real-life” mixed meal. The subjects in the above studies were on mixed or vegetarian diets. Our results disagreed with other studies based on the studied population demographics, ethnicity, co-existing co-morbidities, and type of food or macronutrients offered to subjects; for example, Goettler et al.’s study was conducted only on women with breast cancer [21]. Tsuji’s study differed from ours in two aspects [22]. The ethnicities of the two populations were different, which may play a role in the hormonal response; furthermore, the study evaluated the effect of fat and dietary fiber intake on PRL release, while our study evaluated the effect of a mixed meal on PRL release. The study by Ishizuka et al. included only a small number of men [25]. Carlson et al., Shultz et al., and Velázquez-Villegas et al. all reported on the effect of a protein meal on PRL [26,27,28], which again differed from our approach in evaluating the effect of a mixed meal on PRL release, which may explain the different results.

A further analysis of the results in the gender, age, BMI, and smoking subgroups indicated that PRL levels in females were significantly suppressed after food intake in both the early and postprandial states compared to fasting, while in men, PRL was significantly suppressed after food intake only in the late postprandial state. This was consistent with the results of Coelloa et al. [29], which demonstrated a postprandial prolactin suppression in healthy males. Our results were not consistent with those of Hill et al. [30], which showed that in healthy, non-obese, and obese premenopausal women fed isocaloric breakfasts containing a high carbohydrate or a high fat–protein content, the plasma PRL levels were unaltered by breakfast. The different meal compositions between the two studies may have played a role in the different results.

Our results also indicated that age had a significant effect on the prolactin response to food intake, as subjects younger than 30 years had a significant suppression in both early and late postprandial states compared to fasting, while those older than 30 years showed no significant change in prolactin levels after food intake. Moreover, subjects with a normal BMI showed significant suppression in prolactin levels after food intake, while those overweight and with obesity did not. It was also noted that those who were nonsmokers showed significant suppression after food intake while smokers did not. We could not find previous research that specifically compared the effect of mixed-meal food intake on prolactin levels in obese subjects vs. non-obese, nor in smokers vs. nonsmokers.

### 4.4. BMI and Serum PRL Level

The effect of weight on serum PRL levels was evaluated in several studies with varied results. Roelfsema et al. concluded that basal PRL levels correlated positively with BMI [7]. Wang et al. [31] concluded that serum prolactin levels were positively related to metabolic indexes and disorders in male obese patients. Several other studies did not agree with the results of Roelfsema, including a prospective cross-sectional study conducted by Ernst et al. [17], which found that basal PRL levels were not significantly correlated with the BMI of the subjects after adjusting for the influence of sex. Moreover, after a massive surgically-induced weight loss of about 50 kg, basal serum PRL levels remained completely unchanged, and these levels remained unchanged when data from women and men were analyzed separately. In conclusion, the study by Ernst et al. did not support the notion of a major role of PRL in the pathophysiology of obesity, nor did it support a significant association between basal PRL levels and obesity [17]. The same conclusion was reached in a case–control study conducted by Liu et al. [32] aimed at investigating the association between the PRL serum levels in different obesity phenotypes; metabolically healthy obesity (MHO) and metabolically unhealthy obesity (MUHO). In this study, the MHO group exhibited significantly higher levels of serum PRL than the MUHO group, which was found to have PRL levels similar to the healthy controls.

Birketvedt et al. [33] studied the circadian rhythms of some neuroendocrine peptides including PRL in overweight and normal weight night eating syndrome (NES) subjects and their respective healthy controls. Mean PRL levels tended to be higher in the NES subjects, whereas the overweight subjects tended to have lower PRL levels. However, these effects were only near-significant. In conclusion, Birketvedt et al. did not find any significant correlation between serum PRL levels and BMI. On the other hand, Wagner et al. [18] found that the serum PRL level was associated negatively with BMI and this association remained significant after adjustment for age and gender.

Our results were consistent with those of Ernst et al., Liu et al., and Birketvedt et al. [17,32,33] as the BMI in our analysis did not seem to have a significant effect on the fasting or postprandial PRL levels. As for the study by Wagner et al. [18], our population differed in several aspects, the ethnicity of participants being one of them. In addition, all the subjects recruited by Wagner were overweight or obese with a BMI > 27, with known prediabetes or a family history of diabetes, while 51% of our subjects had a normal BMI and none had known prediabetes.

Our results are also not consistent with Roelfsema et al.’s [7] retrospective study that included postmenopausal women and older men up to age 77 years. Their study was performed in a research unit with a prolactin level measurement every 10 min for 24 h where subjects were not allowed any physical activity except using the restroom, while our study attempted to mimic real-life conditions as described in our Methods above. Wang et al. [31], on the other hand, included patients who were very obese (average BMI 42.8 kg/m^2^), young (average age 24.5 years), and all males, which differed from our population in several ways (Table 1), likely leading to the inconsistency with our results.

### 4.5. Smoking and Serum PRL Level

The effect of smoking on serum PRL levels is still debated. Fuxe et al. documented that PRL secretion is inhibited in chronic cigarette smokers [34]. Coleman et al. demonstrated that nicotine can reduce PRL gene expression in rat pituitary cell lines [35]. Kapoor et al. also reported significant reductions in PRL concentrations in women who smoke during pregnancy and breastfeeding [36].

On the other hand, Blanco-Munoz et al. and Al-Turki et al. reported that the PRL level increases after smoking [37,38]. In addition, Bassey et al. reported no differences in the PRL levels of either active or passive smokers compared to nonsmokers [39]. Garcia-Rizo et al. reported higher PRL levels in newly diagnosed antipsychotic patients compared to controls, and when further analyzed, smoking did not appear to have any significant effect on the PRL levels [40].

Our results did not show any significant difference in PRL levels between smokers and nonsmokers, consistent with the results of Bassey [39] and Garcia-Rizo [40]. The response to food intake was different, as nonsmokers showed a significant suppression of prolactin levels after food intake, while smokers did not. We could not find previous work that investigated the prolactin response to food in smokers compared to nonsmokers.

Limitations include the relatively smaller sample size of older individuals and the age range of the volunteers, which did not include children or adults older than 53 years. Different food choices may have varying effects on serum PRL levels, which we did not analyze because subjects were given their choice of food; however, all subjects were observed to have a mixed meal, and beverages alone like coffee, tea, or juice were not accepted for inclusion. In addition, the effect of sleeping on prolactin levels was not excluded as the actual time of awakening from sleep was not recorded for the subjects on the day of the study. The sexual activities of our subjects before the blood sampling were not reported, which could also limit our results. The pack-year history of smoking was not documented to correlate the effect of the degree of smoking on the prolactin levels of smokers.

## 5. Conclusions

Our study expands on the current knowledge with regard to the effects of gender, age, BMI, food intake, and smoking on serum PRL levels. Our findings suggest that age and gender, as well as the timing of blood sampling relative to meals, may influence the measurement of serum PRL levels obtained in clinical practice. Further research is needed to establish the need for standard reference ranges with attention to age, gender, and the timing of food intake.

## Figures and Tables

**Table 1:** Baseline characteristics of the study subjects.

Variable	n (%)
Sex	
Men	46 (46%)
Women	54 (54%)
Age (years)	
Mean (SD)	29.19 (9.67)
Range	19–53
Age	
<30 years	63 (63)
≥30 years	37 (37)
Smoking status	
No	68 (68)
Yes	32 (32)
BMI	
Normal	51 (51)
Overweight/Obesity	49 (49)
BMI mean (kg/m^2^) (SD)	24.9 (4.6)

**Table 2:** Effect of food intake on prolactin levels among different gender, age, BMI, and smoking groups.

Variable	Serum Prolactin Levels (ng/L)			Pairwise Comparisons (*p*-Value)		
	Fasting T1	1 h Postprandially T2	3 h Postprandially T3			
	Mean (SD)	Mean (SD)	Mean (SD)	T1 vs. T2	T1 vs. T3	T2 vs. T3
**Gender**						
Female	15.35 (8.73)	12.44 (5.33)	11.20 (5.25)	0.001	0.003	0.291
Male	9.30 (5.01)	8.34 (3.40)	7.55 (2.51)	0.187	0.015	0.044
*p*-value (female vs. male)	0.000	0.000	0.000			
**Age**						
≤30 years	15.14 (8.57)	11.88 (5.08)	10.13 (4.67)	<0.001	<0.001	0.002
>30 years	8.29 (3.60)	8.37 (3.98)	8.65 (4.44)	1.00	1.00	1.00
*p*-value (≤30 vs. >30)	0.000	0.003	0.09			
**BMI**						
<25 kg/m^2^	15.14 (9.24)	11.98 (5.49)	10.17 (4.62)	0.002	<0.001	0.006
≥25 kg/m^2^	10.14 (5.21)	9.23 (4.03)	9.01 (4.59)	0.114	0.480	1.00
*p*-value (<25 vs. ≥25)	0.25	0.32	0.86			
**Smoking Status**						
No	13.95 (7.96)	11.47 (5.22)	10.02 (5.02)	<0.001	<0.001	0.046
Yes	9.46 (6.77)	8.50 (3.67)	8.48 (3.22)	0.745	0.822	1.00
*p*-value (no vs. yes)	0.57	0.42	0.89			

**Table 3:** Summary of previous studies’ results showing the food intake effect on serum PRL levels.

Reference Number	Author	Year of Publication	Number of Subjects	Type of Research	EFFECT on Serum PRL Level
19	Plumelle	2014	20	Cohort	No effect from food but decreased during the day
20	Mei	2022	72	Randomized controlled clinical trial	No difference in response to MED/low-carb diet vs. low-fat diet
23	Angelis	2020	157	Epidemiological study	Decrease in response to Mediterranean diet
24	Hill and Wyder	1979	8	Cohort	Vegetarian diet decreased prolactin
21	Goettler	1990	27	Cohort	Significant rise in stage II breast cancer patients
22	Tsuji	2012	393	Cohort	No significant change after intake of unsaturated fat, total fat, dietary fiber
25	Ishizuka	1983	7	Cohort	Increasing levels after food
26	Carlson	1983	14	Cohort	Increased in both men and women after protein intake
27	Schultz	1987	20	Cohort	Increased after dietary energy, protein, total and saturated fatty acids, and oleic acid
28	Velázquez-Villega	2015	Animal study on rats		Increased after consumption of a high-protein/low-carbohydrate diet

## Data Availability

Data unavailable due to privacy or ethical restrictions.
